# Draft Genome Sequence of *Lactobacillus pobuzihii* E100301^T^

**DOI:** 10.1128/genomeA.00185-13

**Published:** 2013-05-09

**Authors:** Chi-ming Chiu, Chi-huan Chang, Shwu-fen Pan, Hui-chung Wu, Shiao-wen Li, Chuan-hsiung Chang, Yun-shien Lee, Chih-ming Chiang, Yi-sheng Chen

**Affiliations:** Department of Biotechnology, Ming Chuan University, Gui-Shan Township, Taoyuan County, Taiwana;; Department of Animal Science, National Chung Hsing University, Taichung, Taiwanb;; Institute of Biomedical Informatics, National Yang-Ming University, Beitou District, Taipei, Taiwanc

## Abstract

*Lactobacillus pobuzihii* E100301^T^ is a novel *Lactobacillus* species previously isolated from pobuzihi (fermented cummingcordia) in Taiwan. Phylogenetically, this strain is closest to *Lactobacillus acidipiscis*, but its phenotypic characteristics can be clearly distinguished from those of *L. acidipiscis*. We present the draft genome sequence of strain *L. pobuzihii* E100301^T^.

## GENOME ANNOUNCEMENT

*Lactobacillus pobuzihii* E100301 is the type strain isolated from a traditional Taiwanese food, pobuzihi (fermented cummingcordia) (1). The species is phylogenetically related to *Lactobacillus acidipiscis* (≤17% relatedness in cDNA sequence of 16S rRNA). *L. pobuzihii* E100301^T^ can produce l-lactic acid from l-arabinose, rhamnose, lactose, and 5-ketogluconate, but not from mannitol. The physiological features of *L. pobuzihii* E100301^T^ are distinct from those of *L. acidipiscis.*

Since this strain is a novel species of the *Lactobacillus* genus, we are intrigued to explore its whole genome and then identify its potential benefits for the fermentation industry. Here, we report the draft genome sequence of *L. pobuzihii* E100301^T^ obtained with the Illumina GAIIx genome analyzer. Short reads (average, 95.6 bases) obtained with 42.05-fold genomic coverage were assembled using *de novo* Velvet algorithms (2) to generate a 2,355,270-bp single chromosome with 67 contigs (53 contigs, >300 bp; 41 contigs, >1 kb; 19 contigs, >40 kb). Close relatives in the public SEED database include *Lactobacillus salivarius* UCC118, *Lactobacillus ruminis* ATCC 25644, and *Lactobacillus plantarum* WCFS1 (Seed Viewer 2.0). A previous study suggests that the species closest to *L. pobuzihii* E100301^T^ is *L. acidipiscis* (1). Because the draft genome sequence of *L. acidipiscis* has not yet been published, the closest neighbor analyzed by the Seed Viewer 2.0 is *L. salivarius* UCC118.

The draft genome sequence comprises 2,250 predicted coding sequences (CDSs), with a mean G+C content of 37.64%. The assembly of contigs for gene finding was performed by GeneMark.hmm with a heuristic approach (3). The putative protein sequences were mapped to Gene Ontology (GO) terms by searching the GO database (4). The CDSs were annotated by searching against the KEGG database. Two hundred eighty-six RAST subsystems are categorized in the genome (5).

Carbohydrate, nucleotide, and amino acid metabolism derivatives are the major types of genes. At least nine genes are involved in three unique metabolic or nutrient transport characteristics of the species, including genes for an arabinose operon repressor (*L. plantarum* WCFS1), l-arabinose isomerase (*L. plantarum* WCFS1), l-rhamnose isomerase (*L. salivarius* ATCC 11741), l-rhamnose 1-epimerase (*L. salivarius* ATCC 11741), l-rhamnose-proton symport (*L. salivarius* UCC118), lactose/cellobiose-specific subunit IIA (*L. ruminis* ATCC 27782), lactose/cellobiose-specific subunit IIB (*Lactobacillus curvatus* CRL 705), a lactose-specific IIBC component (*Lactobacillus casei* BD-II), and lactose-specific transporter subunit IIBC (*Lactobacillus farciminis* KCTC 3681) (strains with homologs used for identification are listed in parentheses). However, only one gene (encoding phosphotransferase system [PTS] family fructose-mannitol porter component IIA [*L. ruminis* SPM0211]) that participates in the transport of mannitol was found in the sequence. Notably, this strain survives under high-salt conditions, and two osmotic-tolerance genes (encoding X-prolyl-dipeptidyl aminopeptidase [*L. salivarius* NIAS840] and proline iminopeptidase [PIP] [*Lactobacillus pentosus* IG1]) were identified. The genes described correspond to the relevant physiological features of the strain.

### Nucleotide sequence accession numbers.

This Whole-Genome Shotgun project has been deposited at DDBJ/EMBL/GenBank under the accession no. APCP00000000. The version described in this paper is the first version, accession no. APCP01000000.
